# Hyperkalemia due to topical timolol for hemangioma

**DOI:** 10.1016/j.jdcr.2023.07.006

**Published:** 2023-07-17

**Authors:** Bushra Alasmari, Abdulaziz Alkhenaizan, Sultan Al-Khenaizan

**Affiliations:** a College of Medicine, King Saud bin Abdulaziz University for Health Sciences, Riyadh, Saudi Arabia; b College of Medicine, Imam Mohammad bin Saud University, Riyadh, Saudi Arabia; c Division of Dermatology, King Abdulaziz Medical City, National Guard Health Affairs, Riyadh, Saudi Arabia

**Keywords:** adverse effect, hemangioma, hyperkalemia, timolol

## Introduction

Infantile hemangioma (IH) is the most common benign vascular neoplasm of infancy, estimated to affect approximately 4% of infants.^1^ The successful use of oral β-blockers for the treatment of IH in 2008 was followed by expanding literature utilizing topical preparations of β-blockers, presuming a safer profile compared to systemic administration.^2^ Topical timolol is a nonselective β-blocker that has been used off-label for treating superficial IH for over a decade, with few reported adverse effects.^2^ Hyperkalemia is an alarming, potentially life-threatening complication of β-blockers, which has been rarely reported with topical timolol when used for IH and glaucoma.^3^^,^^4^ Here, we describe an incident of hyperkalemia in a child given topical timolol eyedrops to treat an ulcerated hemangioma and review the literature on this complication.

## Case report

A 4-month-old female infant was noted to have IH during admission for infantile spasm. She was born at term via normal spontaneous vaginal delivery with a birth weight of 2.6 kg. The IH was present at birth located over the mid-back and has been growing since. On examination, there was a solitary pinkish barely raised plaque over the back measuring 9 × 5 cm. During admission, the patient was started by a colleague dermatologist on timolol maleate 0.5% ophthalmic solution. The mother was instructed to apply it twice daily over the hemangioma, without specific instructions on how many drops to be given. A few days later, routine tests revealed incidental hyperkalemia with an initial potassium level of 5.7 mmol/L, climbing to 6.2 mmol/L. The results were verified through repeated nonhemolyzed blood samples. The patient was asymptomatic with normal vital signs and no electrocardiogram changes. Other biochemical tests including creatinine, blood urea nitrogen, urinalysis, serum sodium, serum osmolality, and blood pH were normal.

Antihyperkalemic measures were immediately initiated with sodium polystyrene sulfonate (kayexalate) 5 mg, twice daily, and sodium bicarbonate 7.5 mEq, twice daily. Concurrently, nephrology and endocrinology teams were involved to investigate and manage the hyperkalemia. Laboratory evaluation showed a normal uric acid level of 201 μmol/L, serum phosphate of 1.9 mmol/L, and serum calcium of 2.55 mmol/L, making tumor lysis syndrome unlikely. A normal renin level of 21.5 μIU/mL, an aldosterone level of 5.1, a normal renin/aldosterone ratio, and a normal adrenocorticotrophic hormone level excluded hypoaldosteronism secondary to adrenal pathology. During her hospital stay, she received cosyntropin 75 unit/m^2^ twice daily, vigabatrin 400 mg, prednisolone 10 mg every 6 hours for 2 weeks, hydralazine 0.5 mg, and omeprazole 5 mg. Due to the unlikely hyperkalemic effect of these medications and their initiation after the onset of hyperkalemia, they were ruled out as a cause of hyperkalemia. Despite thorough evaluation by the involved services, the patient had a protracted course of unexplained hyperkalemia resistant to appropriate treatment given over 3 weeks. Pediatric dermatology service was consulted to follow the hemangioma, and reassessment revealed a 1-cm central ulceration that had developed 1 week prior to the onset of hyperkalemia. We promptly alerted the primary team that timolol is the likely culprit of hyperkalemia and advised to discontinue it immediately. A few days after discontinuation, a decline in potassium level was evident, and hyperkalemia resolved completely in 10 days. The patient was safely discharged with a potassium level of 4.2 mmol/L and remained normokalemic at subsequent follow-up visits.

## Discussion

Guo et al^5^ reported the use of topical timolol for IH for the first time in 2010. Topical timolol is approved by the Food and Drug Administration for the treatment of glaucoma and has been safely used in children and infants for over 30 years.^6^ The reported systemic adverse effects include bradycardia, apnea, and hypothermia.^2^ Hyperkalemia is a very rare adverse effect, which was first described by Swenson in an adult patient using timolol eyedrops for glaucoma who developed hyperkalemia after initiating oral prednisolone and resolved upon discontinuation of timolol eyedrops.^3^ In 2018, Pandhi et al^4^ reported hyperkalemia with electrocardiogram changes in a 3-month-old infant following topical timolol for IH as a part of posterior fossa malformations, hemangioma, arterial anomalies, coarctation of the aorta/cardiac defects, and eye abnormalities syndrome. Similar to Swenson, the infant was also on oral prednisolone prior to developing hyperkalemia. However, our patient developed hyperkalemia before starting prednisolone for infantile spasm.

The mechanisms of hyperkalemia with β-blockers are not fully understood, with many possible explanations.^7^ Some of the proposed mechanisms include interference with intracellular potassium uptake, suppression of renin secretion, and induction of endothelial cell apoptosis; releasing potassium extracellularly.^7^ This could be exacerbated by the catabolic effects of prednisolone, resulting in a high potassium load and possibly contributing to the protracted course of hyperkalemia as in our patient.

Being lipophilic, timolol is approximately 8-10 times more potent than propranolol in its β-receptor blocking activity, with a half-life of 5 hours and a duration of activity reaching up to 24 hours.^8^ Drolet et al^9^ found timolol in the plasma of infants receiving timolol topically for IH at concentrations known to exert β-blocking activity in adults. Although not demonstrated in their study, the potential of increased absorption in ulcerated hemangiomas or those near mucosal surfaces has been a concern for many authors.^10^

In conclusion, topical timolol has been proclaimed to be a safer alternative to oral propranolol for treating small IH with a low risk of systemic toxicity.^2^ This case report adds to the previously reported possible risk of hyperkalemia with topical timolol.^4^ Until pharmacokinetic data and guidelines are established, we advocate for cautious use of topical timolol and keeping a high index of suspicion for systemic adverse effects. We agree with McMahon et al^10^ that topical timolol is better avoided in ulcerated hemangioma or those near mucosal site, and the dose should be limited to one or two drops per application.

## Conflicts of interest

None disclosed.

## References

[1] Munden A., Butschek R., Tom W.L. (2014). Prospective study of infantile haemangiomas: incidence, clinical characteristics and association with placental anomalies. Br J Dermatol.

[2] Lin Z., Zhang B., Yu Z., Li H. (2020). The effectiveness and safety of topical β-receptor blocker in treating superficial infantile haemangiomas: a meta-analysis including 20 studies. Br J Clin Pharmacol.

[3] Swenson E.R. (1986). Severe hyperkalemia as a complication of timolol, a topically applied ß-adrenergic antagonist. Arch Intern Med.

[4] Pandhi D., Jakhar D., Tandon A., Singal A. (2018). Topical timolol in PHACES syndrome: is it safe?. Indian J Dermatol Venereol Leprol.

[5] Guo S., Ni N. (2010). Topical treatment for capillary hemangioma of the Eyelid using β-blocker solution. Arch Ophthalmol.

[6] Coppens G., Stalmans I., Zeyen T., Casteels I. (2009). The safety and efficacy of glaucoma medication in the pediatric population. J Pediatr Ophthalmol Strabismus.

[7] Al-Rwebah H., Alkhodair R., Al-Khenaizan S. (2020). Propranolol-induced hyperkalemia in the management of infantile hemangioma. JAAD Case Rep.

[8] Cimolai N. (2019). Neuropsychiatric adverse events from topical ophthalmic timolol. Clin Med Res.

[9] Drolet B.A., Boakye-Agyeman F., Harper B. (2020). Systemic timolol exposure following topical application to infantile hemangiomas. J Am Acad Dermatol.

[10] McMahon P., Oza V., Frieden I.J. (2012). Topical timolol for infantile hemangiomas: putting a note of caution in “cautiously optimistic.”. Pediatr Dermatol.

